# Single-stranded RNA viruses activate and hijack host apical DNA damage response kinases for efficient viral replication

**DOI:** 10.1007/s42764-022-00064-3

**Published:** 2022-02-28

**Authors:** Pengcheng Li, Chenchen Xu, Xiaoyan Zhang, Cheng Cao, Xuejuan Wang, Gang Cai

**Affiliations:** 1 The First Affiliated Hospital of USTC, MOE Key Laboratory for Membraneless Organelles and Cellular Dynamics, Division of Life Sciences and Medicine, University of Science and Technology of China, Hefei, 230001 Anhui China; 2 College of Life Science and Resources and Environment, Yichun University, Yichun, 336000 China; 3 Beijing Institute of Biotechnology, 27 Taiping Rd, Haidian District, Beijing, 100850 People’s Republic of China; 4 CAS Center for Excellence in Molecular Cell Science, Chinese Academy of Sciences, Hefei, 230026 China

**Keywords:** DNA damage response, ATM, ATR, RNA virus replication

## Abstract

The ataxia-telangiectasia mutated (ATM) and ATM-Rad3-related (ATR) are apical kinases that orchestrate the multifaceted DNA damage response (DDR) to a variety of genotoxic insults and regulate genomic stability. Whether RNA virus also manipulates the host’s DDR machine to facilitate replication is largely unknown. In this study, we revealed that single-stranded RNA virus replication specifically elicits host ATM- and ATR-mediated pathway activation and boosts their expression. The activated ATM and ATR are hijacked to the virus replication factory in the cytoplasm and facilitate viral gene expression and replication. Specific inhibitors targeting ATM and ATR strikingly block the viral proliferation and replication and inhibit expression of virus proteins. Our results reveal a novel, or otherwise noncanonical, conserved function of ATM/ATR outside DDR in promoting the replication of single-stranded RNA virus and provide an important mechanism of host–pathogen interactions.

**Supplementary Information:**

The online version contains supplementary material available at 10.1007/s42764-022-00064-3.

Dear Editor,

A variety of exogenous and endogenous factors have constantly caused extensive DNA damage and genomic instability. DNA damage response (DDR) is a complex protein modification regulatory network, which detects DNA damage, signals its presence and mediates its repair (Jackson & Bartek, 2009). DDR has an impact on a wide range of cellular events, are biologically significant because they prevent diverse human diseases. The ataxia–telangiectasia mutated (ATM) and ATM-Rad3-related (ATR) are apical kinases that orchestrate the multifaceted DDR to a variety of insults and regulate genomic stability (Lee & Paull, 2021). Notably, In yeast and human, ATM is not essential, whereas ATR is (Waterman et al., 2020).

During infection, most viruses have evolved their means to evade DDR surveillance and harbor complex interactions with cellular DDR pathways. It is well established that DNA viruses manipulate the host DDR machinery to optimize their replication processes (Mertens & Knipe 2021). Whether RNA virus also manipulates the host’s DDR machine to facilitate replication is unknown. At present, only a few RNA viruses have been reported to interplay with some DDR factors (Ariumi et al., 2008; Ren et al., 2020; Xu et al., 2011). Recently, an antiviral drug screen identifies ATR kinase inhibitor as potent blocker of SARS-CoV-2 replication, which also inhibit replication of SARS-CoV-1 and the Middle East respiratory syndrome coronavirus (MERS-CoV) as well (Garcia et al., 2021). Our long-term research focus on the ATM and ATR kinases (Qiu et al., 2019; Wang et al., 2016; Wang et al., 2017; Xin et al., 2019) prompted us to check whether ATM and/or ATR play a critical role in RNA virus replication. To unravel the putative molecular interplays between single-stranded RNA viruses’ replication and the host ATM and ATR kinases, we selected the in vitro replication model system of the Porcine Reproductive and Respiratory Syndrome Virus (PRRSV) and Zaire Ebolavirus (EBOV), which are the positive- and negative-sense single-stranded RNA viruses, respectively.

First, we examined whether specific inhibitors of ATM and ATR kinases affected the replication of PRRSV virus in Marc-145 cells. We treated Marc-145 cells infected with PRRSV virus with several different ATM and ATR inhibitors with variant doses at distinct stages of virus infection (Supplementary information, Fig. S1). It was found that ATM inhibitor (KU55933) and ATR inhibitor (VE821) can significantly inhibit the formation of progeny and reduce the viral titer, which is more evident at the late replication state (Fig. 1a). Moreover, ATM inhibitor (KU55933) and ATR inhibitor (AZD6738) have a synergistical effect on inhibiting PRRSV replication (Fig. 1a and Supplementary information, Fig. S2). We examined the inhibitors effects on the abundance of ATM and ATR and the expression of virus nucleocapsid (N) protein at 0 h, 2 h, 16 h and 24 h post-infection (h.p.i.) and found that both ATM and ATR inhibitors could significantly inhibit the expression of viral N protein and significantly reduce the abundance of ATM, but had little effect on the abundance of ATR (Fig. 1b and Supplementary information, Fig. S2). These findings suggest that the replication of PRRSV virus in Marc-145 cells critically depends on the kinase activities of ATM and ATR.

**Fig. 1:**
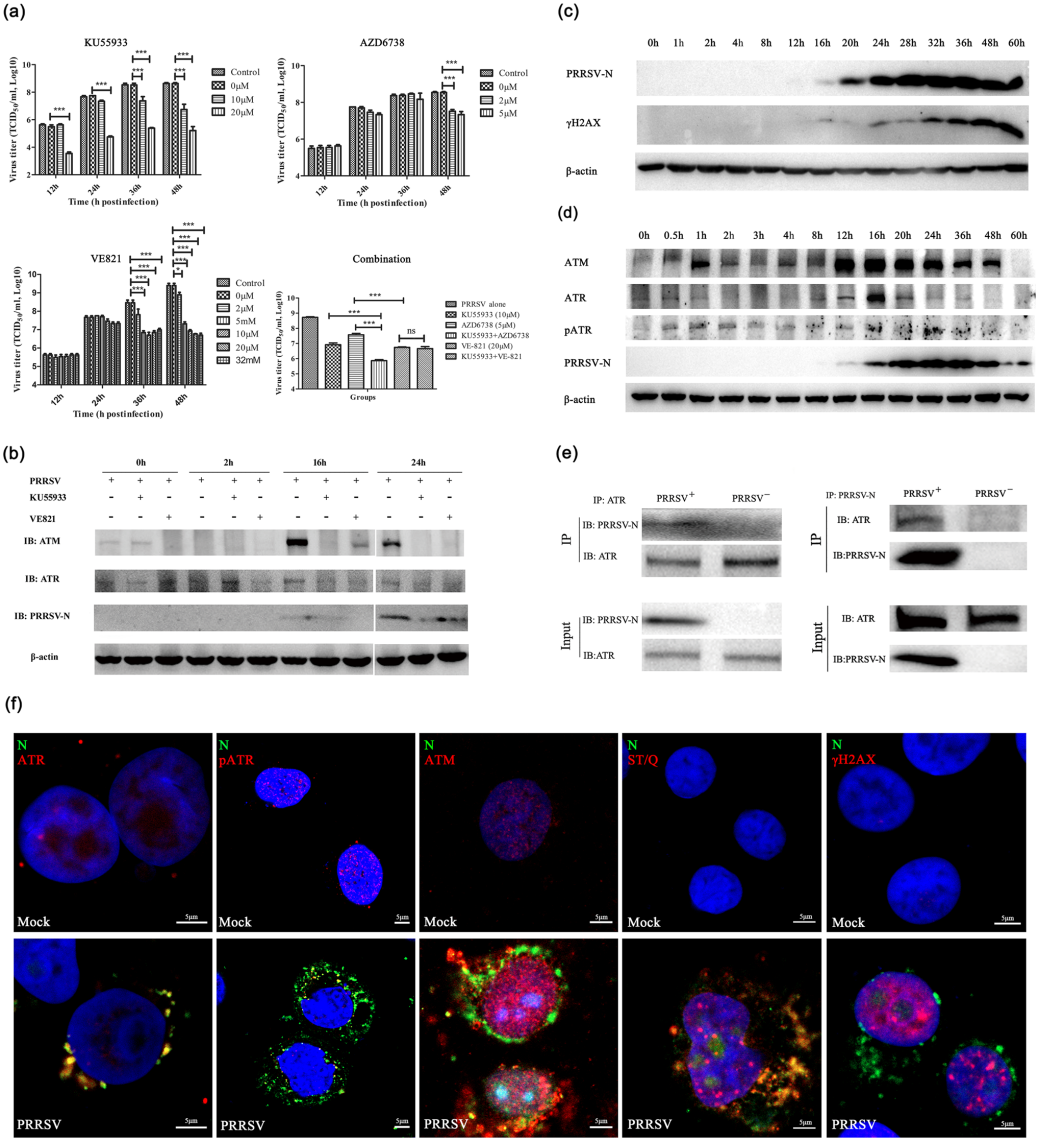
ATM and ATR promote PRRSV replication in MARC-145 cells. **a** Effects of ATM, and ATR inhibitors on PRRSV replication in MARC-145 cells. MARC-145 cells were pretreated with different working concentrations of KU55933, AZD6738 and VE-821 for 2 h prior to PRRSV infection (MOI 0.5). As a control, cells were infected with the same dose of PRRSV without the inhibitor treatment. To determine if there were a synergistic effect between ATMi and ATRi during PRRSV infection, KU55933 and AZD6738 or VE-821 were added simultaneously in MARC-145 cells. PRRSV titers were determined on MARC-145 cells as TCID50 based on the Reed-Muench method at a different indicated timepoint. Statistical significance was evaluated by determining *p*-values. ns, *p* > 0.05; *p < 0.05; **p < 0.01, ***p < 0.001. **b** Protein expression levels of specific DNA damage key sensor detected using phospho-specific antibodies ant- H2AX (Ser139) by western blot analysis, along a 60 h time-course of PRRSV-infected MARC-145 cells (MOI of 0.5). Increased levels of H2AX phosphorylated forms were detected, starting at 8 h post-infection (hpi). PRRSV N protein expression was used as control for the viral infection time-course. β-actin expression was used as a loading control. **c** PRRSV infection elicits ATR and ATM pathway. Western blot analyses of ATM, ATR, phosphorylated ATM, phosphorylated ATR and γH2AX in PRRSV-infected MARC-145 cells are shown. MARC-145 cells were infected with PRRSV (MOI 0.5) at the indicated timepoints. Cells were lysed and analyzed by Western blot with specific antibodies. β-actin served as a loading control. Viral replication was confirmed by Western analysis of N protein with anti-PRRSV N monoclonal antibodies. **d** ATM or ATR inhibition by specific inhibitors disrupt PRRSV protein synthesis in MARC-145 cells. Inhibition of ATR kinase activity induced by VE-821 at 20 µmM or ATM kinase activity induced by KU55933 at 10 µmM modulated PRRSV protein synthesis. Expression levels of a N protein were severely reduced. Mock-infected cells were used as a control group in immunoblot analysis. **e** The interaction between ATR and PRRSV was analyzed by Co-immunoprecipitation. The lysate was subjected to immunoprecipitation with ATR or PRRSV N protein antibody, respectively. **f** PRRSV alters nuclear localization of ATR and ATM kinases hijacks the phosphorylated ATR to promote virus replication. pATR distribution pattern displayed nuclear enlarged accumulations (red) in ASFV infected cells (green). DAPI stained nuclear DNA (blue). Mock-infected was used as negative control for ATR activation in immunofluorescence studies. Representative confocal images showing that PRRSV infection in MARC-145 cells. MARC-145 cells were mock-infected or infected with PRRSV (MOI 0.5). Cells were fixed and immunostained with specific antibodies at 48 hpi. Red represents ATR, pATR, ATM, ST/Q (phospho-ATM/ATR substrate) and γH2AX, green represents PRRSV(N), and blue (DAPI) represents cell nucleus. Scale bars = 5 μm

In response to DNA damage, ATM and ATR are activated and phosphorylate multiple substrates, including the H2AX histone variant (γH2AX), themselves (pATM and pATR), to regulate cell cycle progression, replication fork stability, and DNA repair (Waterman et al., 2020). We are curious about whether PRRSV replication will also result in γH2AX, pATM and pATR during time-course infection. We detected the expression and phosphorylation of related proteins at different stages of virus infection by Western blotting. It was found that with the increase of the expression of PRRSV N protein, the phosphorylation level of H2AX increased, and the expression of ATM and ATR also increased basically synchronously (Fig. 1c and d). Among them, the expression of ATM increased significantly, whereas ATR expression was only marginally increased. The ATR phosphorylation level increased in the early stage of virus infection, maintained a high phosphorylation level during the whole infection stage, and further increased at 16–36 h.p.i., which was coincident with the high-level expression of N protein (Fig. 1d). The ATM phosphorylation level did not change significantly (data not shown). Through the co-IP experiments, we also found that PRRSV N protein and host ATR kinase could be reproducibly and reciprocally co-precipitated from cell lysate, indicating there was perhaps a direct interaction between the ATR kinase and the PRRSV N protein (Fig. 1e).

We next asked whether ATM and ATR were directly involved in the replication process of PRRSV. Under normal physiological state and cellular DNA damage conditions, ATM and ATR mainly function in the nucleus. Through indirect immunofluorescence experiment (Supplementary information, Fig. S3 and Table S1), we observed the expression and localization of ATM and ATR kinase underwent significant changes during virus replication (Fig. 1f). In contrast with the mock experiment without PRRSV infection, the abundance of ATM and ATR kinases were most notably increased, and the majority of ATR proteins were sequestrated outside the nucleus, especially in the nucleus periphery region. Especially, ATR and its phosphorylated form were specifically associated with the viral replication center. ATM expression increased most significantly, almost evenly distributed among the nucleus and nucleus periphery, but not colocalized with the PRRSV replication center. Corroborated with Western blotting observation, the phosphorylation level of H2AX and ATR were significantly increased. In addition, the ATR and its activated form-pATR were markedly hijacked to the viral replication center. These observations illuminate that ATM and ATR are directly involved in the PRRSV replication. In particular, ATR kinase is specifically sequestrated by PRRSV to its replication center, phosphorylates a series of downstream substrates, and creates an environment conducive to viral replication.

To elucidate whether the mechanism of ATM and ATR regulating viral replication is conserved, and we explored whether ATM and ATR also play a critical role in RNA negative strand virus-EBOV replication. We found that the expression of VP35 protein of EBOV harbored the same trend as H2AX phosphorylation, ATM expression and ATR phosphorylation (Fig. 2a and b). During EBOV replication, the expression of ATR basically did not change, and the ATM phosphorylation increased significantly only in the late stage of replication. Indirect immunofluorescence also observed that EBOV replication induced extensive phosphorylation of H2AX and ATR, and the effect of ATM phosphorylation was insignificant (Supplementary information, Fig. S4 and Table S1). Most ATM, ATR and phosphorylated ATR in cells are specifically sequestrated by EBOV to its replication center, where extensive phosphorylation of ATM and ATR downstream substrates occurred (Fig. 2c). Such phosphorylation should be mainly catalyzed by the activated and phosphorylated ATR. In general, PRRSV and EBOV virus replication have the same effects on ATM/ATR abundance, self-phosphorylation and phosphorylation of downstream ATM/ATR substrates, obviously increasing ATM expression and ATR phosphorylation. ATM and ATR are hijacked to the virus replication center and rely on the kinase activity to facilitate viral replication.

**Fig. 2:**
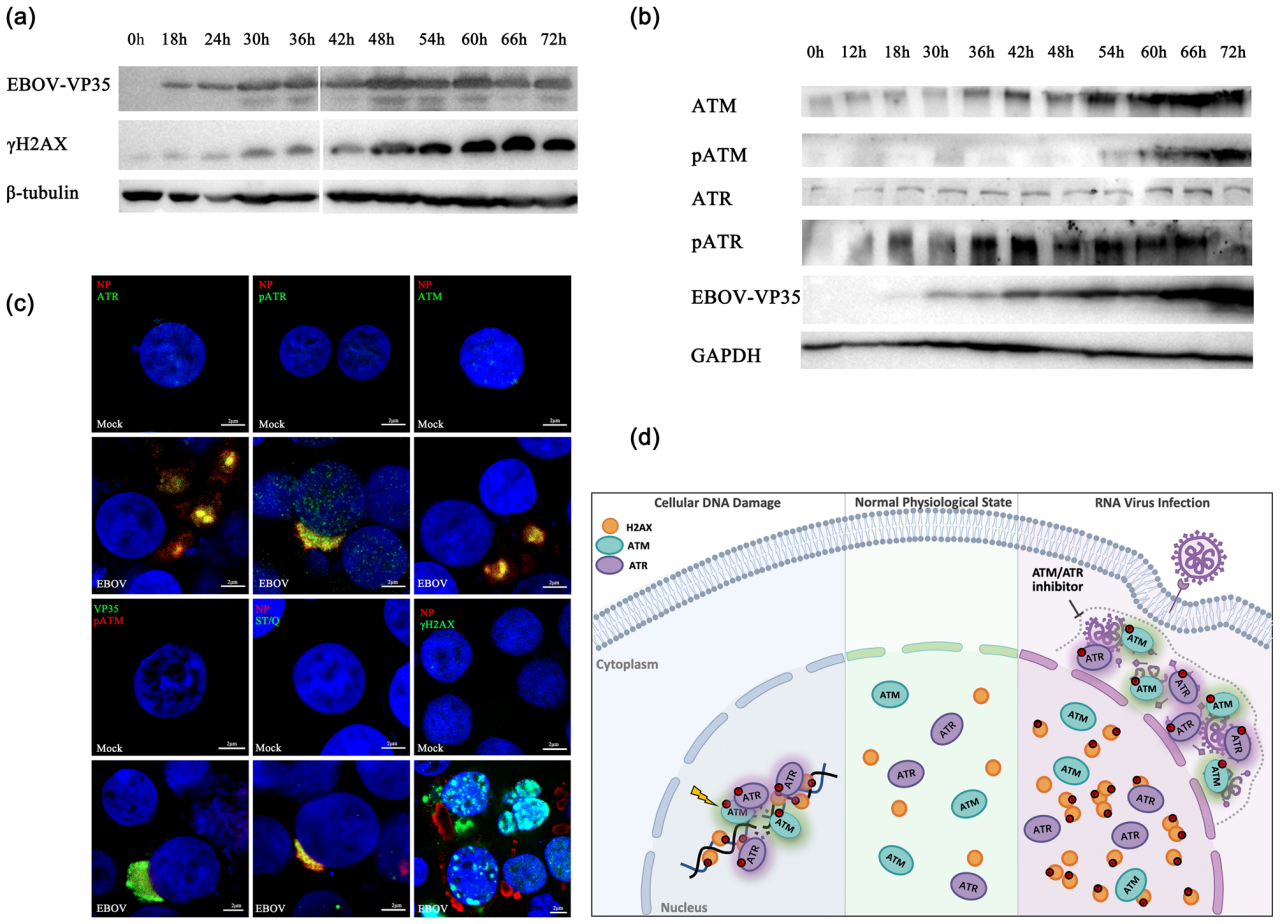
ATM and ATR promote EBOV replication in HEK293 cells. **a**, **b** Western blot analyses of ATM, ATR, phosphorylated ATM, phosphorylated ATR and γH2AX in EBOV-infected HEK293 cells are shown. HEK293 cells were transfected with plasmids encoding the EBOV minigenome assay components (NP, VP35, VP30, L, the EBOV-specific minigenome, and T7 polymerase) at the indicated timepoints. Cells were lysed and analyzed by Western blot with specific antibodies. β-tubulin and GAPDH served as the loading control. Viral replication was confirmed by Western analysis of VP35 protein with anti-EBOV VP35 monoclonal antibodies. **c** EBOV infection induced phosphorylation of ATR, ATM and H2AX and hijacked ATM, ATR, pATM, pATR and their phosphorylated substrates to facilitate virus replication. Representative confocal images showing that EBOV infection in HEK293 cells. HEK293 cells were mock-infected or infected with EBOV (transfected with plasmids encoding the EBOV minigenome assay components). At 48 h post-transfection, cells were fixed and immunostained with specific antibodies. Green represents ATR, pATR, ATM, EBOV(VP35), ST/Q (phospho-ATM/ATR substrate) and γH2AX, red represents pATM and EBOV(NP), and blue (DAPI) represents cell nucleus. Scale bars = 2 μm. **d** Schematic of the RNA virus activate and hijack host ATM and ATR kinases to facilitate viral replication. Under normal physiological state, ATM and ATR mainly function in the nucleus. In response to DNA damage, ATM and ATR are recruited to the DNA damage sites, activated and phosphorylate multiple substrates to initiate the DNA damage checkpoints. RNA virus replication induces a significant change of the expression and localization of ATM and ATR, both kinases are activated and hijacked to the virus replication center in the cytoplasm, and responsible for many ATM/ATR downstream substrates’ phosphorylation, which is required for efficient viral replication. Specific inhibitors targeting ATM and ATR significantly inhibit the replication of RNA viruses

In summary, our study has revealed novel roles for apical ATM and ATR kinases in the cellular DNA damage response to RNA virus infection and elucidate the redundant mechanism for ATM and ATR activation to promote efficient virus replication (Fig. 2d). Both ATM and ATR kinase are activated by RNA virus infection and hijacked to the virus replication center in the cytoplasm, and responsible for many ATM/ATR downstream substrates’ phosphorylation. Both ATM and ATR activation promote replication, and inhibition of these kinases activity substantially reduce replication. Collectively, our results reveal a novel, or otherwise noncanonical, conserved function of ATM/ATR outside DDR in promoting the replication of single-stranded RNA virus and provide an important mechanism of host–pathogen interactions. Our improving understanding of DNA-damage responses in RNA virus replication hold the promise to provide new avenues for treatment of RNA viral infectious diseases.

## Supplementary Information

Below is the link to the electronic supplementary material.

Supplementary file1 (DOCX 2232 KB)

## References

[CR1] Ariumi Y, Kuroki M, Dansako H (2008). The DNA damage sensors ataxia-telangiectasia mutated kinase and checkpoint kinase 2 are required for hepatitis C virus RNA replication. Journal of Virology.

[CR2] Garcia G, Sharma A, Ramaiah A (2021). Antiviral drug screen identifies DNA-damage response inhibitor as potent blocker of SARS-CoV-2 replication. Cell reports.

[CR3] Jackson SP, Bartek J (2009). The DNA-damage response in human biology and disease. Nature.

[CR4] Lee JH, Paull TT (2021). Cellular functions of the protein kinase ATM and their relevance to human disease. Nature Reviews Molecular Cell Biology.

[CR5] Mertens ME, Knipe DM (2021). Herpes simplex virus 1 manipulates host cell antiviral and proviral DNA damage responses. mBio.

[CR6] Qiu S, Liu S, Zaoti ZF, Wang X, Cai G (2019). Modulation of fatty acid synthase by ATR checkpoint kinase Rad3. Journal of Molecular Cell Biology.

[CR7] Ren S, Ur Rehman Z, Gao B (2020). ATM-mediated DNA double-strand break response facilitated oncolytic Newcastle disease virus replication and promoted syncytium formation in tumor cells. PLoS pathogens.

[CR8] Wang X, Chu H, Lv M (2016). Structure of the intact ATM/Tel1 kinase. Nature Communications.

[CR9] Wang X, Ran T, Zhang X (2017). 3.9 Å structure of the yeast Mec1-Ddc2 complex a homolog of human ATR-ATRIP. Science.

[CR10] Waterman DP, Haber JE, Smolka MB (2020). Checkpoint responses to DNA double-strand breaks. Annual review of biochemistry.

[CR11] Xin J, Xu Z, Wang X, Tian Y, Zhang Z, Cai G (2019). Structural basis of allosteric regulation of Tel1/ATM kinase. Cell Research.

[CR12] Xu LH, Huang M, Fang SG, Liu DX (2011). Coronavirus infection induces DNA replication stress partly through interaction of its nonstructural protein 13 with the p125 subunit of DNA polymerase delta. The Journal of Biological Chemistry.

