# Supplementary material for: Single-stranded RNA viruses activate and hijack host apical DNA damage response kinases for efficient viral replication
Source: Genome Instab Dis. 2022 Feb 28;3(2):83–7. doi: 10.1007/s42764-022-00064-3 (PMC8883245; doi:10.1007/s42764-022-00064-3)
Supplement: Supplementary file 1 — Supplementary file1 (DOCX 2232 KB) [file 42764_2022_64_MOESM1_ESM.docx]

**Materials and methods**

**Cell culture, virus, reagents, and antibodies**

MARC-145, an African Green Monkey kidney cell line highly permissive to PRRSV infection, which was obtained from the American Type Culture Collection (ATCC, Rockville, MD, USA), was used for PRRSV propagation and antiviral assays. HEK293, a human embryonic kidney cell line was used for EBOV minigenome assays. MARC-145 and HEK293 cells were cultured in Dulbecco’s modified Eagle’s medium (DMEM, Gibco, USA) supplemented with 10% (v/v) heat-inactivated fetal bovine serum (FBS, Gibco, USA) at 37°C in a humidified 5% CO_2_ incubator. PRRSV NJ strain was kindly provided by National Research Center of Engineering and Technology for Veterinary Biologicals (Jiangsu Academy of Agricultural Science, Nanjing, China) and propagated in MARC-145 cells. HEK293 cells and expression plasmids for EBOV minigenome assays were kindly provided by Prof. Cheng Cao, Beijing Institute of Biotechnology (27 Taiping Rd, Haidian District, Beijing, China).

ATM kinase inhibitor (ATMi, KU55933) and ATR kinase inhibitors (ATRi, AZD6738 and VE-821) were purchased from Selleck Chemicals Company (Houston, TX, USA). Stock solutions of these inhibitors were prepared in dimethyl sulfoxide (DMSO). All reagents were stored at −70°C and diluted to the desired working concentration in fresh medium immediately before use. The RIPA buffer and phenylmethyl sulfonylfluoride (PMSF) were purchased from Beyotime Institute of Biotechnology (Beyotime, China). The transfect reagent Fugene HDwas purchased from Promega and used following the manufacturer's instructions.

Mouse anti-PRRSV N protein monoclonal antibody was prepared in our laboratory. Anti-ATR(C1) (sc-515173 AF594) was purchased from Santa Cruz Biotechnology. Anti-ATM (NB100-104), anti-phospho-ATM (Ser 1981) (NB100-306), and anti-phospho-ATR (Thr1989) (NBP2-43564) were purchased from Novus Biologicals. Anti-phospho-ATM (Ser 1981) (4526), anti-phospho-ATR (Ser 428) (2853), anti-phospho-Histone H2AX (Ser 139) (9718), anti-phospho-(Ser/Thr) ATM/ATR Substrate (2909) were purchased from Cell Signaling Technology.

Anti-β-actin was purchased from Absin (Shanghai, China). Anti-β-tubulin was purchased from Abmart Shanghai Co., Ltd. (Abmart, China). Anti-GAPDH was purchased from Yeasen Biotechnology Shanghai Co., Ltd. (Yeasen, China). Secondary antibodies, including horseradish peroxidase-conjugated (HRP) goat anti-rabbit or goat anti-mouse IgG antibodies (Abmart, China) were validated for use in Western blot analysis. The goat anti-mouse and goat anti-rabbit Alexa Fluor secondary antibodies, including Alexa Fluor 488 and 594 (molecular probes) used for indirect immunofluorescence, were purchased from Abmart Shanghai Co.,Ltd. (Abmart, China).

**Cytotoxicity assay for ATM and ATR inhibitors**

On the day before the experiment, seed 5 x 10^4^ MARC-145 cells/well in a 96-well plate that achieve a sub-confluent (75%) monolayer the day of the experiment. Cells were treated with serial dilution of inhibitors, permitting a range of 8 concentrations. On the day of the experiment, prepare inhibitor dilutions in tissue culture medium. For MARC-145 cells, a range of 0 to 128 μM for base analogs (KU55933, AZD6738) were used. MARC-145 cells were exposed to the several concentrations of inhibitors for 24 h. Cell viability was performed by Cell Counting Kit-8 (CCK-8; Dojindo, no. CK04-20). According to the manufacturer’s instructions, 10 µL of thawed CCK-8 solution was added into 100 µL fresh Dulbecco’s modified Eagle’s medium into each well, and the plates were incubated for 2 h at the same incubator conditions. The graph was prepared according to the absorbance value, which was read at 450 nm.

**Effect of ATM and ATR inhibitors on viral infection**

In order to investigate whether ATM and ATR inhibitors affect PRRSV infection, MARC-145 cells were treated with a mixture of nontoxic concentrations of the inhibitors and PRRSV [multiplicity of infection (MOI) 0.5] at 37°C for 48h. As a control, cells were infected with the same dose of PRRSV without the inhibitors. Subsequently, the antiviral efficacy was evaluated by analysis of virus loads. PRRSV titers were determined on MARC-145 cells as the median tissue culture infective dose (TCID_50_) based on the classical Reed–Muench method.

**The dose screening experiment of ATM and ATR inhibitors against PRRSV infection in MARC-145 cells**

MARC-145 cells were seeded into 6-well and grown to confluency. Cells were preincubated with ATM and ATR inhibitors at a series of concentrations in 2% (v/v) serum DMEM for 2 h at 37°C, and then PRRSV (MOI 0.5) was added to each well and incubated for 1 h at 37°C. After removing the unattached viruses by washing with cold DMEM, the cells were cultured in in 2% serum DMEM at 37 °C for 48h. The MARC-145 cells were subjected to three freeze-thaw cycles in preparation for measuring virus loads.

**PRRSV association assay**

The association assays were investigated on MARC-145 cell monolayers in 6-well plates. Tissue culture medium was replaced with antibiotic-free medium 1 day before viral infection. Different types of experiments were performed. Pretreatment, coincubation, and post-treatment of PRRSV-infected MARC-145 cells with and without the ATM/ATR inhibitors were evaluated. MARC-145 cells were preincubated with the inhibitors for 2 h at 37°C, and then PRRSV (MOI 0.5) was added to each well and incubated for 1 h at 37°C. After removing the unbound viruses by washing with cold DMEM, the cells were incubated at 37°C for 48 h. The inhibitors and PRRSV (MOI 0.5) were mixed and co-incubated simultaneously with MARC-145 monolayers for 48 h at 37°C. PRRSV (MOI 0.5) was added to MARC-145 monolayers and incubated for 1 h at 37°C before challenging with the inhibitors; the plates were then incubated for 48 h at 37°C. The MARC-145 cells were subjected to three freeze-thaw cycles in preparation for measuring virus loads in the three assays. All assays were performed in triplicate on three consecutive cell passages.

MARC-145 cells were pretreated with a working concentration of KU55933, AZD6738, or VE-821 for 2 h prior to PRRSV infection (MOI 0.5). As a control, cells were infected with the same dose of PRRSV without the inhibitor treatment. PRRSV titers were determined as described above at a different indicated timepoint.

**Viral infection and drug treatment**

MARC-145 cells were seeded into 24-well or 6-well plates and grown to confluency. PRRSV (MOI 0.5) was added to each well and incubated for 1 h at 37°C. After removing the unbound viruses by washing with cold DMEM, the cells were cultured at 37°C for a different time. As a control, cells were preincubated with the inhibitors for 2 h at 37°C. The cell samples were collected for IFA and Western blot analysis. All assays were performed in triplicate on three consecutive cell passages.

**EBOV Minigenome assay**

EBOV minigenome assay was performed according to the established protocol from Cao Lab, Beijing Institute of Biotechnology, with some modifications. Briefly, HEK293 cells were seeded into 6-well plates and grown to confluency, and then transfected with optimized amounts of expression plasmids, i.e., 125 ng pCAGGS-NP, 125 ng pCAGGS-VP35, 75 ng pCAGGS-VP30, 1000 ng pCAGGS-L, 250 ng tetrascistronic minigenome expression plasmid, and 250 ng pCAGGS-T7 (for initial transcription of the minigenome), using 5.5 μL transfection reagent per well. At 24 h after transfection, the medium was exchanged against 5% serum DMEM. The cell samples were collected at the different indicated timepoints after transfection for IFA and Western blot analysis.

**Extraction of host proteins**

MARC-145 and HEK293 cells were washed twice with ice-cold phosphate-buffered saline (PBS, pH 7.2–7.4) and then lysed with an RIPA buffer (1% Triton X-100, 1% deoxycholate, 0.1% SDS, 150 mM NaCl, 50 mM Tris pH 7.4, 10 mM EDTA pH 8.0 dissolved in PBS), containing phosphatase and protease inhibitors (1 mM phenyl-methyl-sulfonyl-fluoride, 1 mM Na_3_VaO_4_, 25 mM NaF) incubated on ice for 30 min. Cell lysates were centrifuged for 5 min at 14 000 ***g*** and the supernatants were aliquoted and kept at –70°C. In addition, cytoplasmic proteins were collected by nuclear extraction kit (Solarbio Science & Technology Co, China) according to the manufacturer’s instructions. The protein concentrations were determined by a bicinchoninic acid assay protein determination kit (Multi Sciences Biotech Co, China).

**Western blot analysis**

The prepared protein samples were subjected to 4–15% SDS–polyacrylamide gel electrophoresis (SDS-PAGE) and transferred onto a nitrocellulose blotting membrane (GE Healthcare Life Science, Amersham, Protran 0.45 NC, Germany). The membranes were then blocked with 5% skimmed milk in TBST (10 mM Tris-HCl, pH 7.5, 150 mM NaCl, 0.1% Tween 20) for 1 h at room temperature. The membranes were inoculated with primary antibodies overnight at 4°C. After washing three times with TBST (10 min/each), the membranes were incubated with HRP-conjugated secondary antibodies at room temperature for 1 h. Monoclonal mouse anti-β-actin, anti-GAPDH and anti-β-tubulin were used to monitor sample loading. The washing procedure was repeated, and specific signals were detected with the Super Signal West Pico chemiluminescent substrate (Pierce Biotechnology, Rockford, IL) using a multi-chemiluminescence image analysis system (Tanon 5200, Tanon, Guangzhou, China). The intensities of protein bands were quantified using Image J.

**Indirect immunofluorescence assay (IFA)**

MARC-145 cells were differentiated on glass coverslips which were placed in 24-well tissue culture plates until each well was 75% confluent. MARC-145 were infected with PRRSV for the indicated time. HEK293 cells were differentiated on glass coverslips, which were placed in 6-well tissue culture plates until each well was 75% confluent. HEK293 cells were transfected with all EBOV minigenome components and collected. The cell preparations were fixed for 15 min at room temperature in PBS-4% paraformaldehyde. The fixed cells were then permeabilized with 0.3% (v/v) Triton X-100 in PBS for 15 min at room temperature, washed thrice with PBS, blocked with 1% bovine serum albumin at 37°C for 30 min, inoculated with a primary antibody overnight at 4°C, and washed three times again with PBS. Antibody binding was detected using secondary antibodies conjugated with Alexa Fluor 488, or 594 for 1 h in a moist container in the dark at 37°C. The cells were then stained with DAPI (0.1 μg/ml) for 4 min at room temperature. Finally, the coverslips were mounted on the microslides and air dried. Images were visualized and captured on a confocal fluorescence microscope (ZEISS 710, 980, Germany).

**Immunoprecipitation**

MARC-145 cells were infected with PRRSV (MOI 0.5) for 24 h. Cells were washed twice with ice-cold PBS and then lysed with the RIPA buffer containing phosphatase and protease inhibitors (1 mM phenyl-methyl-sulfonyl-fluoride, 1 mM Na_3_VaO_4_, 25 mM NaF) incubated on ice for 30 min. After sonication, cell lysates were centrifuged for 5 min at 14 000 ***g***. Cell extract was mixed with anti-ATR, anti-PRRSV NP antibody at 4°C overnight. Agarose beads were added at a ratio of 1 mg of extract per 120 μl of agarose at 4°C for 2 h. The beads were then pelleted at 2 500 ***g*** for 3 min and washed with lysis buffer five times. The beads were directly boiled in SDS loading buffer.

**Statistical analyses**

Statistical analyses were performed using GraphPad Prism software (Graph Pad Software, Inc., San Diego, CA, USA). All values are representative of the mean of at least three independent experiments. Results were expressed as means ± SEM. ANOVA and unpaired Student’s t-test were employed to determine statistical differences among multiple groups. Statistical significance was evaluated by determining *p*-values. ns, *p* > 0.05; *, *p* < 0.05; **, *p* < 0.01, ***, *p* < 0.001.





**Figure S1. Cytotoxicity assay for ATM and ATR inhibitors.**

Indicated concentrations of KU55933, AZD6738 and VE-821 were added to MARC-145 cells for 24 h. Cell viability was performed by CCK-8 assay. The relative cell viability was calculated as ratios in treated cells relative to control cells, and the values of the control cells were set to 100%.


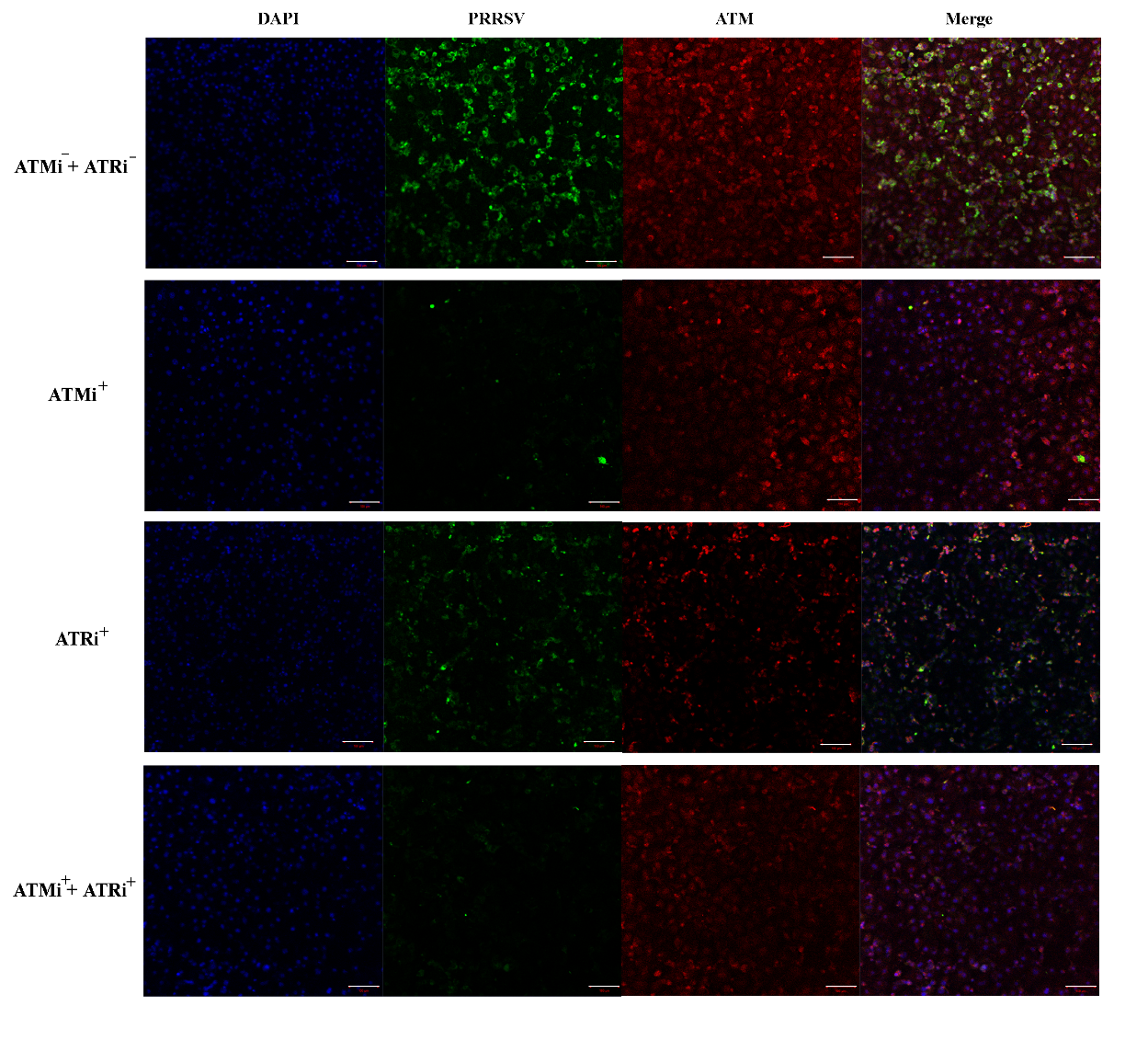


**Figure S2.** **The inhibitory effect of ATMi and ATRi during PRRSV infection was observed by IFA.**

MARC-145 cells were pretreated with ATMi (KU55933, 10 μM) or/and ATRi (AZD6738, 5 μM) for 2 h prior to PRRSV infection (MOI 0.5). As a control (mock), cells were infected with the same dose of PRRSV without the inhibitor treatment. At 48 h post-infection, MARC-145 cells were fixed for IFA. Red represents ATM, green represents PRRSV, and blue (DAPI) represents cell nucleus. Scale bars = 100 μm.

**
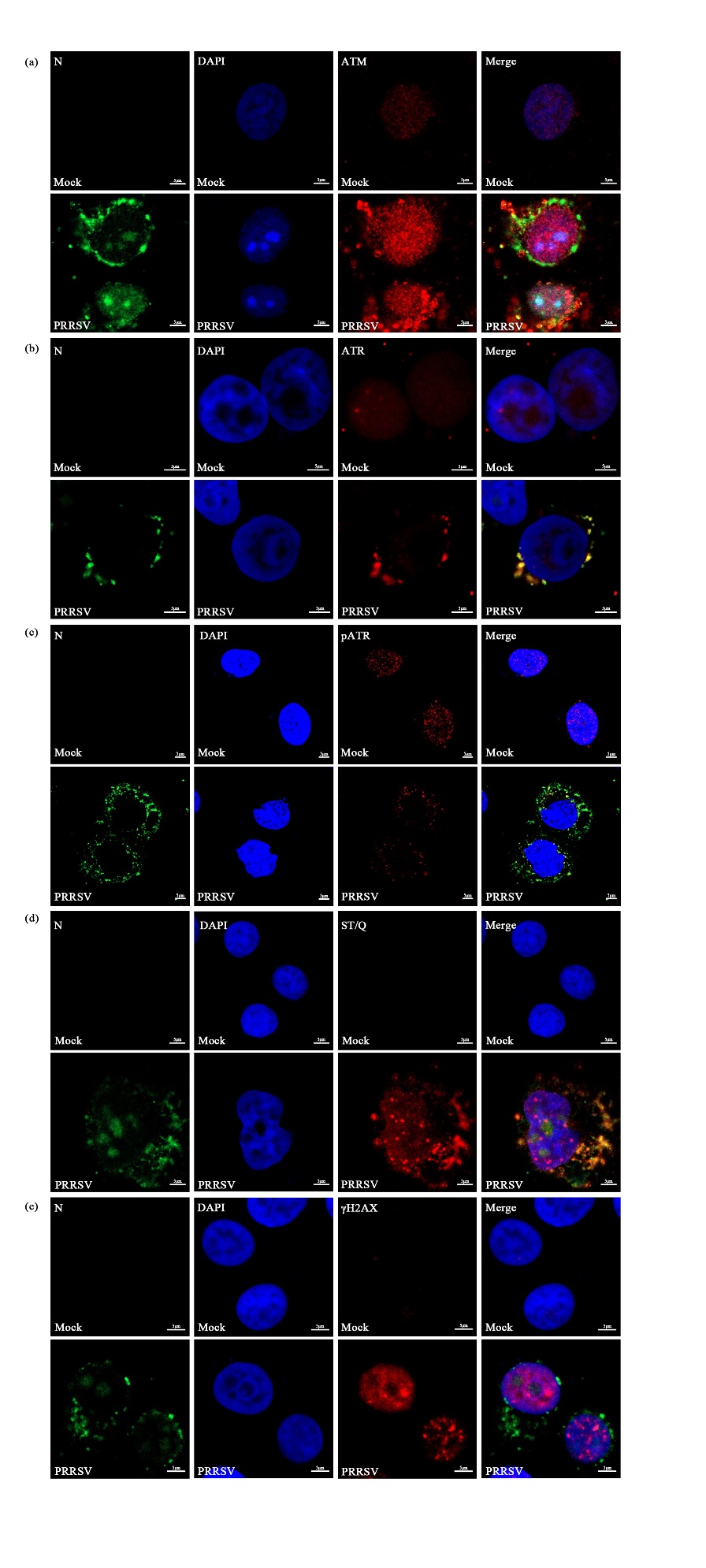
**

**Figure S3. Representative confocal images of PRRSV infection in MARC-145 cells.**

MARC-145 cells were mock-infected or infected with PRRSV (MOI 0.5). At 48 h post-infection, cells were fixed and immune-stained with specific antibodies. (a) ATR. (b) pATR. (c) ATM. (d) ST/Q (phospho-ATM/ATR substrate). (e) γH2AX. Scale bars = 5 μm.


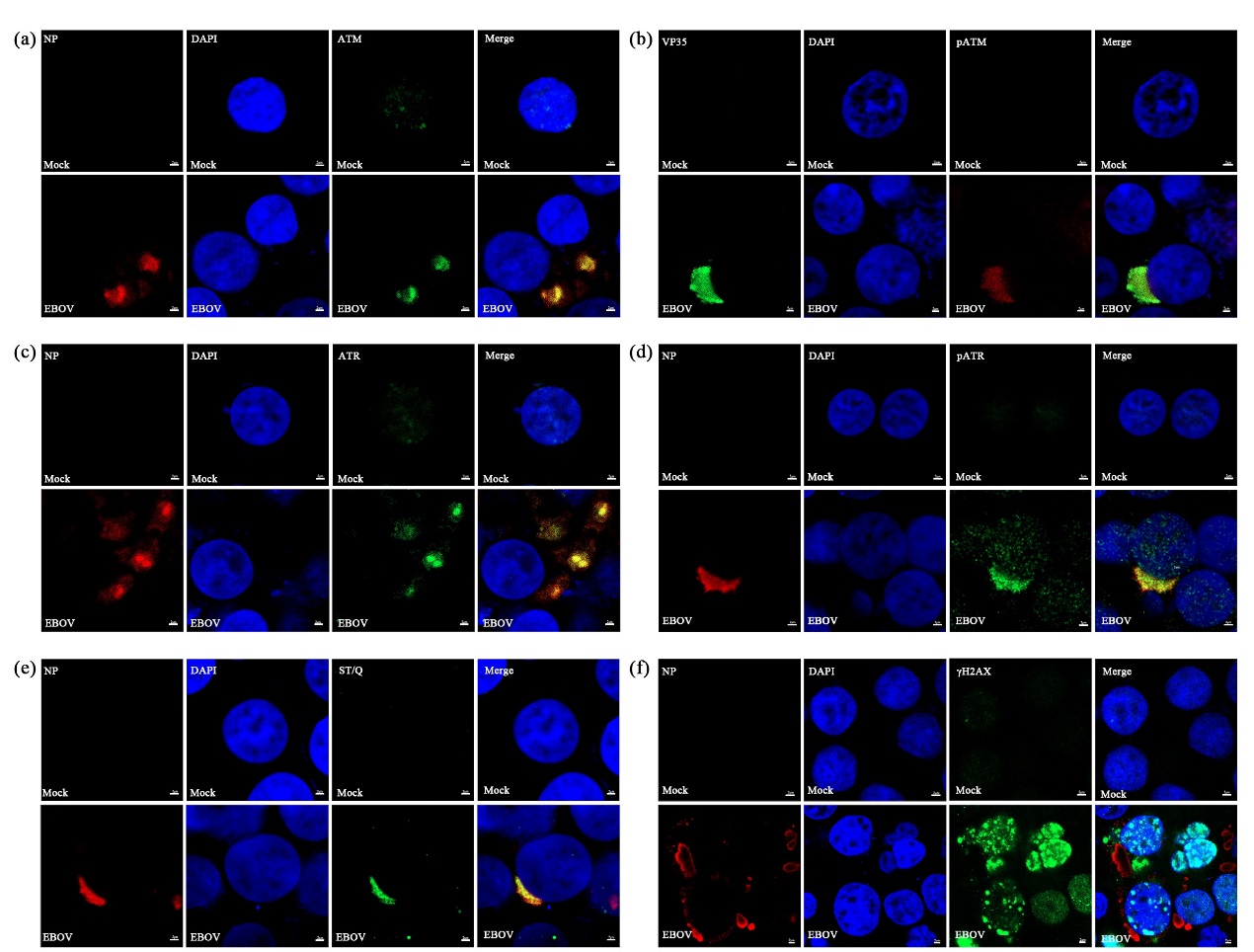


**Figure S4. Representative confocal images of EBOV infection in HEK293 cells.**

HEK293 cells were mock-infected or infected with EBOV (transfected with plasmids encoding the EBOV minigenome assay components). At 48 h post-transfection, cells were fixed and immune-stained with specific antibodies. (a) ATM. (b) pATM. (c) ATR. (d) pATR. (e) ST/Q (phospho-ATM/ATR substrate). (f) γH2AX. Scale bars = 2 μm.

**Table S1. Colocalization analyses of ATM, ATR, phosphorylated ATM, phosphorylated ATR and ST/Q with PRRSV or EBOV.**

| Region-Region | ScatterQuadrant | Pearson | Manders |
| --- | --- | --- | --- |
| PRRSV-ATM | Colocalization | 0.00811 | 0.91451 |
| PRRSV-ATR | Colocalization | 0.61338 | 0.93812 |
| PRRSV-pATR | Colocalization | 0.1309 | 0.89377 |
| PRRSV-ST/Q | Colocalization | 0.40312 | 0.90033 |
| EBOV-ATM | Colocalization | 0.6338 | 0.94902 |
| EBOV-pATM | Colocalization | 0.10802 | 0.9479 |
| EBOV-ATR | Colocalization | 0.58529 | 0.96635 |
| EBOV-pATR | Colocalization | 0.26241 | 0.93241 |
| EBOV-ST/Q | Colocalization | 0.39342 | 0.92806 |
